# *In vivo* study and thermodynamic investigation of two lanthanum complexes, La(dpp)_3_ and La(XT), for the treatment of bone resorption disorders†
†Electronic supplementary information (ESI) available: Speciation diagrams for metal–ligand complexes (Fig. S1 and S3), ITC analysis of H_5_XT-hydroxyapatite titration (Fig. S2), plasma concentrations of La(dpp)_3_ and La(XT) following IV dose of 1 mg kg^–1^ per day taken at beginning & end of trial (ng g^–1^ weight, mean ± SD, *n* = 6) (Table S1). See DOI: 10.1039/c5sc01767j


**DOI:** 10.1039/c5sc01767j

**Published:** 2015-08-03

**Authors:** J. F. Cawthray, D. M. Weekes, O. Sivak, A. L. Creagh, F. Ibrahim, M. Iafrate, C. A. Haynes, K. M. Wasan, C. Orvig

**Affiliations:** a College of Pharmacy and Nutrition , University of Saskatchewan , 104 Clinic Place , Saskatoon , SK S7N 2Z4 , Canada . Email: kishor.wasan@usask.ca; b Medicinal Inorganic Chemistry Group , Department of Chemistry , University of British Columbia , 2036 Main Mall , Vancouver , BC V6T 1Z1 , Canada . Email: orvig@chem.ubc.ca; c Faculty of Pharmaceutical Sciences , University of British Columbia , 2146 East Mall , Vancouver , BC V6T 1Z3 , Canada; d Michael Smith Laboratories and Department of Chemical and Biological Engineering , University of British Columbia , Vancouver , BC V6T 1Z4 , Canada; e Pfizer Inc. , Eastern Point Road , Groton , CT 06340 , USA

## Abstract

Bone density diseases such as osteoporosis affect a significant number of people worldwide. Lanthanide ions are functional mimics of calcium ions, able to substitute for Ca^2+^ in the bone mineral component, hydroxyapatite (HAP). Bone undergoes a continuous remodelling cycle and lanthanides can affect this cycle, exerting a positive influence on bone mineral. We have been engaged in efforts to find new lanthanide containing complexes as active agents for treatment of these diseases and have identified two lead compounds, 3-hydroxy-1,2-dimethylpyridin-4(1*H*)-one (Hdpp) and a phosphinate-EDTA derivative, bis[[bis(carboxymethyl)amino]-methyl]phosphinate (H_5_XT). In this paper, we report *in vivo* data for the first time for the two lead compounds. The pharmacokinetics of La(dpp)_3_ suggest the complex is rapidly cleared from plasma. We demonstrate that La^3+^ accumulates in the bone following IV dose of either La(dpp)_3_ or La(XT) and we have investigated the influence of each chelating ligand on the incorporation of La^3+^ into HAP using ITC and HAP-binding studies.

## Abstract 2

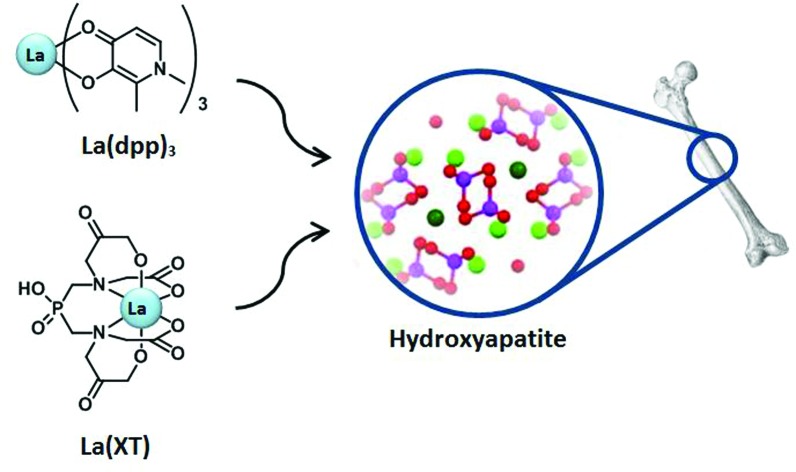
Lanthanum could act as a preventative measure against bone resorption disorders; two compounds are thoroughly investigated both *in vivo* and *ex vivo* as potential oral drug candidates.

## Introduction

Bone density disorders such as osteoporosis are well-established diseases that affect 1 in 5 men, as well as 200 million women worldwide, with 1 in 3 women over 50 experiencing osteoporotic fractures.^1^,^2^ Bone is a porous structure that is constantly being remodelled, with the process of bone formation by osteoblasts and bone resorption by osteoclasts being tightly regulated under normal conditions. Any imbalance within the remodelling process leads to bone resorption diseases such as osteoporosis, characterised by decreased bone mass and poor bone quality. This leads to increased risk of fracture, in the order of 40% in developed countries, and can lead to serious complications in the elderly. The morbidity and mortality associated with osteoporosis gives rise to high personal and financial costs.

To date, the most useful method for the prevention and treatment of osteoporosis is through pharmaceutical intervention with bisphosphonates. Bisphosphonates are the synthetic mimic of the pyrophosphate moiety found in bone mineral and act by inhibiting the resorption of bone by inactivating osteoclasts. Whilst being effective forms of treatment for osteoporosis, there are a number of significant disadvantages of bisphosphonates such as unwanted side-effects. Oral bisphosphonates such as alendronate can lead to upper gastrointestinal problems; patients must take the medication on an empty stomach, at the same time each morning and must stay fully upright for 30 minutes after therapeutic ingestion. Such stringent dosing regimens often lead to poor patient compliance, reducing intestinal absorption and the efficacy of the medicine. As bisphosphonates accumulate in the bone, their safety in long-term use has recently been questioned by several researchers, including the Food and Drug Administration (FDA).^3^,^4^

Hydroxyapatite (HAP) is the main mineral component of bone. Both biological and synthetic HAP crystallise in the hexagonal form having the *P*6_3_/*m* space group and including two formula units per cell, each with 44 atoms and a Ca/P ratio of 1.67.^5^,^6^ An important property of HAP is its ability to undergo substitution of both cations and anions.^7^–^11^ It is interesting to note that within the HAP unit cell there are two crystallographically distinct calcium sites, Ca(1) and Ca(2), with some cations displaying preferential substitution for one site over the other.^12^–^14^

Lanthanides, including the 14 4f-block elements as well as lanthanum, are considered bone-seekers as they are known to have a high affinity for bone due to their strong interaction with the inorganic phosphate in hydroxyapatite.^15^,^16^ Many lanthanides exhibit physicochemical similarities with calcium. For instance, trivalent lanthanum and divalent calcium share similar ionic radii and donor atom preferences but the higher charge on La^3+^ can lead to a high affinity for Ca^2+^ sites in biological molecules.^17^–^19^ Bone is constantly being remodelled through formation of new bone by osteoblasts and resorption by osteoclasts. This can lead to substitution of Ca^2+^ ions with La^3+^ within bone and, through this continuous remodelling cycle of bone, lanthanides can affect cellular activity and potentially exert a positive influence on bone mineral.^20^,^21^

Lanthanum, in the form of lanthanum carbonate (La_2_(CO_3_)_3_, Fosrenol™) is currently used to treat hyperphosphatemia, a condition caused by elevated phosphate levels in the blood. There is a demonstrated dose-dependent accumulation of lanthanum in bone with long retention times.^22^–^24^ Lanthanum influences the bone histology and bone-resorption activity of osteoclasts *in vitro*.^25^–^27^ Therefore lanthanum has been proposed as a potential preventative measure for osteoporosis; however, its low bioavailability requires that high doses be administered, leading to adverse gastrointestinal tract side effects.^28^ Altering the chemical environment around the La(iii) ions through use of chelating ligands has the potential to mitigate these adverse effects and improve both the oral bioavailability of La(iii) and the bone-targeting ability. Bisphosphonates, the current preferred treatment of bone resorption diseases, show a high affinity for HAP. Ln(iii) does as well, and its incorporation into an appropriate chelating ligand could serve to more selectively target the Ln(iii) to the bone.^29^

**Fig. 1:**
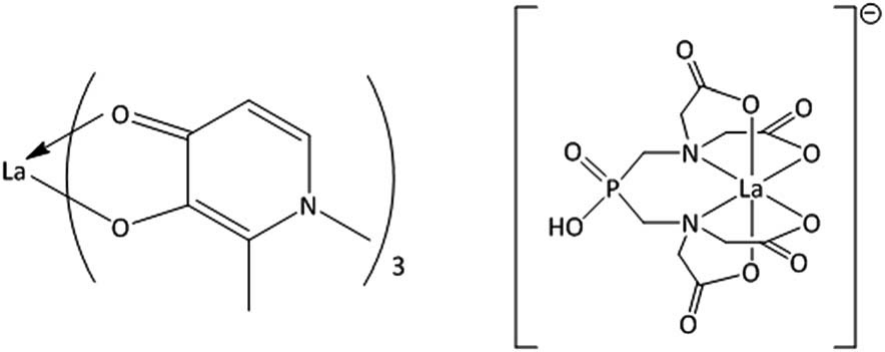
Structures of the metal–ligand complexes formed between La^3+^ and Hdpp (La(dpp)_3_, left) and H_5_XT (La(XT), right), the two lead compounds in this study.

We have therefore engaged in an ongoing project that explores the use of lanthanide complexes for the treatment of osteoporosis and other bone density disorders.^30^–^32^ Our goal has been to identify suitable chelators of lanthanum having the potential to increase the bioavailability of the metal ion while reducing unwanted side effects by lowering the concentrations required and controlling the delivery of the metal. Based on solubility parameters, lipophilicity, hydroxyapatite binding studies, cellular uptake, and cytotoxicity, we have identified two compounds possessing the desired properties: 3-hydroxy-1,2-dimethylpyridin-4(1*H*)-one (Hdpp) and a phosphinate-EDTA derivative, bis[[bis(carboxy-methyl)amino]methyl]phosphinate (H_5_XT) (Fig. 1). When compared to the clinically used lanthanum carbonate, the complexation of La by Hdpp improves cellular uptake, shows low cellular toxicity (EC_50_ > 100 μM in MG-63 cells) and may improve the oral bioavailability of the metal ion *in vivo*. Chelators containing phosphonate groups are already used to target radionuclides to bone for diagnostic or therapeutic purposes^33^–^36^ and the presence of the phosphinic acid group on H_5_XT can be expected to have a similar bone-targeting ability. We have previously investigated the ion-exchange of a series of lanthanides with hydroxyapatite and quantified the thermodynamics of this ion-exchange process by isothermal titration calorimetry (ITC).^37^

In this paper we expand upon the biological studies of Barta^30^ and Mawani^31^ and report *in vitro* time-dependent studies of La^3+^ binding to hydroxyapatite, along with equilibrium binding constants (*K*_a_s) determined by ITC. We also report for the first time *in vivo* data for the two lead compounds, La(dpp)_3_ and La(XT), including the pharmacokinetics and tissue distribution of La(dpp)_3_ following a single dose to rats, as well as tissue and bone distribution of La^3+^ following multiple doses of La(dpp)_3_ and La(XT).

## Results and discussion

### Plasma and tissue clearance and pharmacokinetics of La(dpp)_3_

**Fig. 2:**
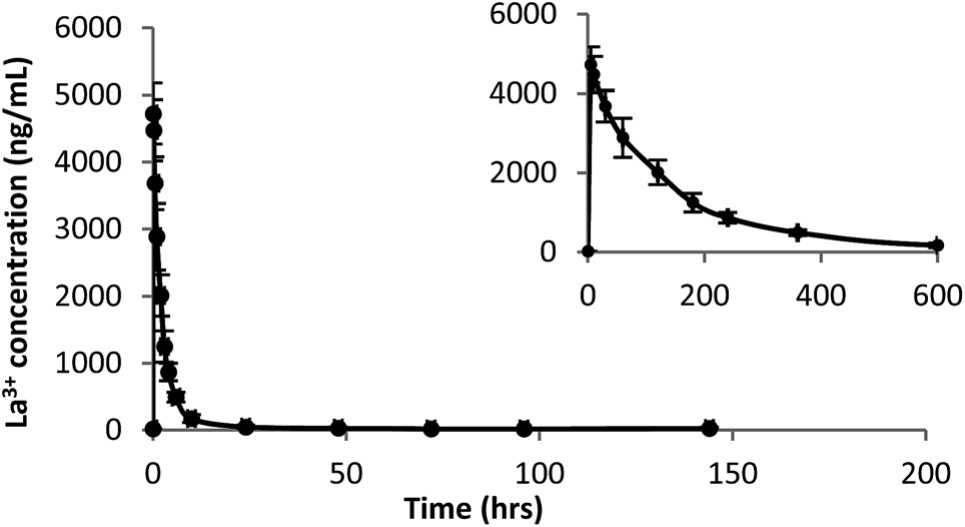
Plasma La^3+^ concentration time (h) profile following single intravenous dose (1 mg kg^–1^) of La(dpp)_3_ in SD rats. Inset shows expansion of plasma concentration time profile from 0 to 600 minutes.

**Fig. 3:**
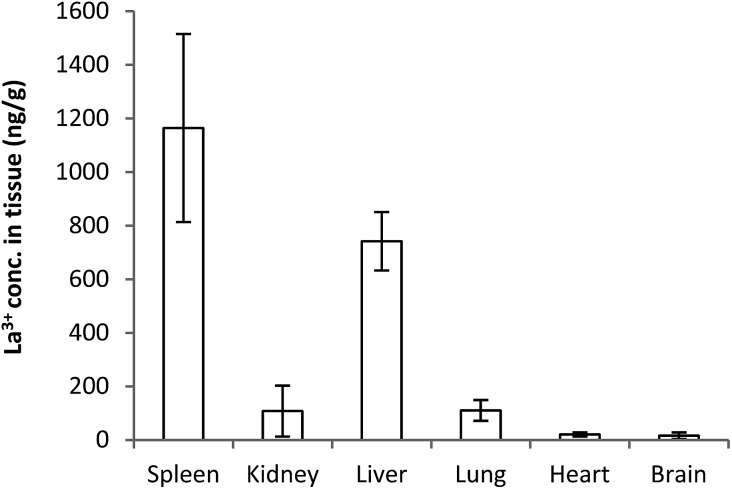
Tissue distribution of lanthanum after 5 days following a single intravenous dose of 1 mg kg^–1^ of La(dpp)_3_.

A plot of La^3+^ plasma concentration *versus* time from Sprague Dawley (SD) rats (*n* = 6) administered La(dpp)_3_ intravenously at a dose of 1 mg kg^–1^ at time 0 h is shown in Fig. 2. The corresponding La^3+^ concentration in organs analysed by ICP-MS following this are shown in Fig. 3. The pharmacokinetic parameters derived by non-compartmental analysis are shown in Table 1. When administered as the complex, La(dpp)_3_, plasma levels of lanthanum show a peak (*C*_0_) of 4973 ± 557 ng mL^–1^ which rapidly decreased to approximately 10% of *C*_0_ within 6 hours and back to pre-dose concentrations (∼15 ng mL^–1^) within 24 hours. Clearance of La^3+^ from plasma was 77 mL h^–1^ kg^–1^ and the steady state volume of distribution was 265 mL kg^–1^. Lanthanum from La(dpp)_3_ was not detected in the plasma beyond 10 h, showing a mean residence time of 4 h.

**Table 1:** Pharmacokinetic parameters of the lanthanide complex derived by non-compartmental analysis (*n* = 6; mean ± SD) following intravenous administration of La(dpp)_3_ (1 mg kg^–1^) in SD rats

Parameter	Unit	Mean	SD
*K*_el_	h^–1^	0.20	0.04
*T*_1/2_	h	3.61	0.85
*C*_0_	ng mL^–1^	4973.09	556.75
AUC_0–∞_	h ng mL^–1^	13 155.37	1812.52
Cl	mL h^–1^ kg^–1^	77.24	10.73
AUMC_0–∞_	h H ng mL^–1^	45 778.12	12 609.75
MRT	h	3.46	0.69
*V*_ss_	mL kg^–1^	265.41	51.33

Whilst plasma levels of La^3+^ above pre-dose concentrations were not detected after 24 h, La^3+^ levels were still detected up to the last sampling point (144 h) in the organs (Fig. 3). The concentration is significantly higher for spleen and liver (1164 ± 350 & 741 ± 109 ng g^–1^, respectively) whereas the heart (20 ± 7 ng g^–1^) and brain (15 ± 12 ng g^–1^) have the lowest levels. It is interesting to note the relatively low concentration of La^3+^ in the kidney (108 ± 95 ng g^–1^), significantly less than for the liver and spleen. This is consistent with previous studies indicating the liver is the main organ of excretion for lanthanum and the kidneys play a negligible role in its elimination following intravenous doses of La_2_(CO_3_)_3_.^38^–^40^

### Tissue distribution of La(dpp)_3_ and La(XT) following multiple doses

**Fig. 4:**
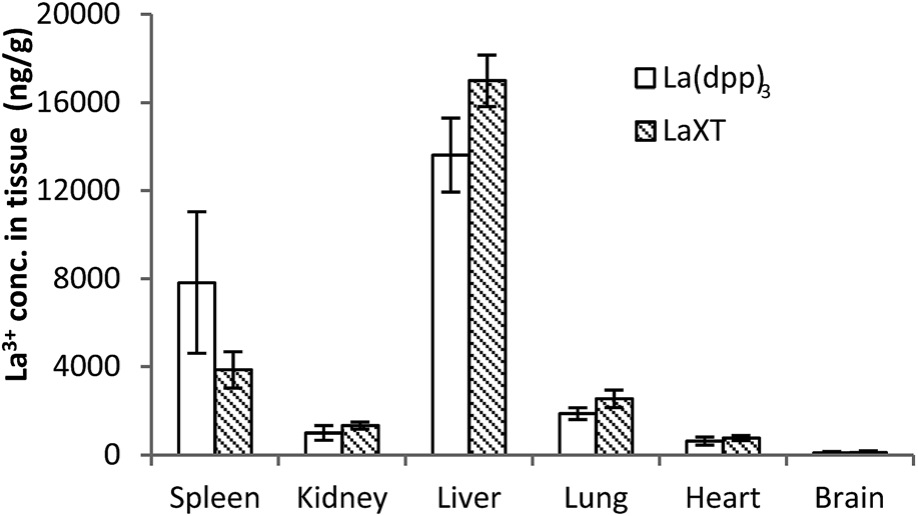
Tissue distribution of the lanthanum from the complexes, La(dpp)_3_ and La(XT), after multiple intravenous dose administrations at 1 mg kg^–1^ per day for 5 days in SD rats (*n* = 6; mean ± SD).

**Fig. 5:**
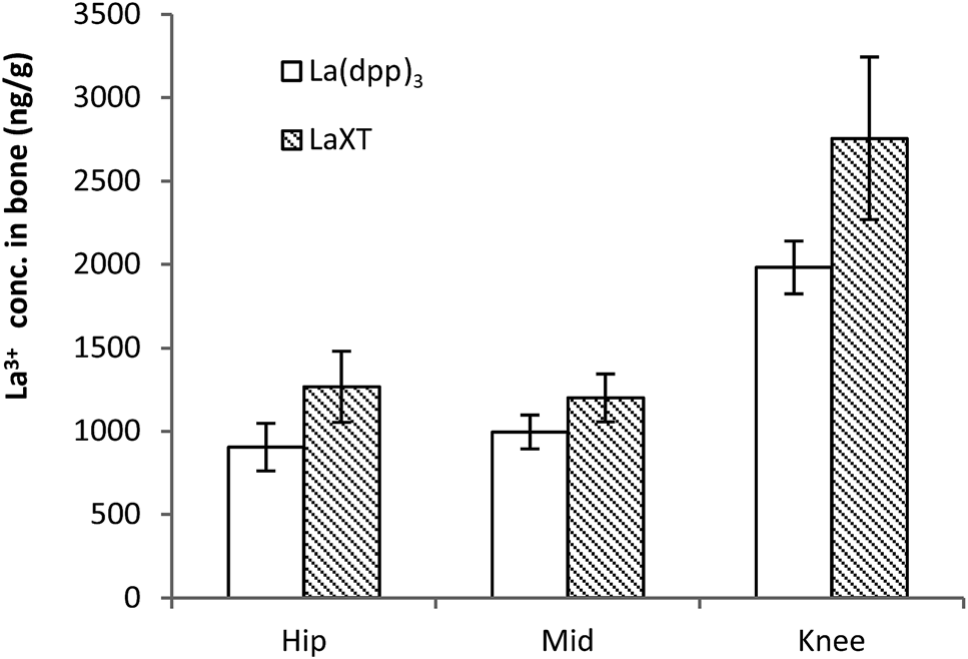
Bone (femur) distribution of lanthanum from the complexes La(dpp)_3_ and La(XT) after multiple doses of 1 mg kg^–1^ per day for 5 days in SD rats (*n* = 6; mean ± SD).

The distribution of lanthanum in organs and bone in SD rats (*n* = 6) following the administration of multiple intravenous doses of either La(dpp)_3_ or La(XT) are shown in Fig. 4 (tissue) and Fig. 5 (bone). The first point to note is that the organ distribution of La^3+^ is similar for both La(dpp)_3_ and La(XT). The highest concentration of La^3+^ following multiple doses with either chelate complex was in the liver (13 611 ± 1687 ng g^–1^ for La(dpp)_3_, 16 983 ± 1160 ng g^–1^ for La(XT)) and spleen (7817 ± 3211 ng g^–1^ for La(dpp)_3_, 3854 ± 827 ng g^–1^ for La(XT)), similar to what was found following a single intravenous dose of La(dpp)_3_. Again, the lowest accumulation was in the heart (635 ± 178 ng g^–1^ for La(dpp)_3_, 768 ± 115 ng g^–1^ for La(XT)) and the brain (101 ± 60 ng g^–1^ for La(dpp)_3_, 112 ± 84 ng g^–1^ for La(XT)), with only relatively low concentration of La^3+^ detected in the kidney (992 ± 321 ng g^–1^ for La(dpp)_3_, 1320 ± 157 ng g^–1^ for La(XT)). Plasma levels after 5 days of treatment were similar for both La(dpp)_3_ and La(XT) (5517 ± 1188 ng g^–1^ and 5999 ± 302 ng g^–1^), respectively.

No significant difference in metal-ion distribution was recorded between the two complexes except for differences in animal-to-animal variation in tissue and plasma concentrations. From Fig. 4 and Table S1,† larger inter-animal variability is noted for La(dpp)_3_. A possible cause of the variability may lie with the differences in thermodynamic stability of the complex and/or its kinetic inertness *in vivo*. The difference in denticity of the two chelating ligands investigated will influence the stability and speciation of the metal ion as discussed later.

From Fig. 5 the bone distribution of lanthanum appears slightly greater in animals treated with La(XT) compared to La(dpp)_3_. Statistical analysis of the datasets for uptake of lanthanum derived from either La(dpp)_3_ or La(XT) in the hip, mid and knee sections of bone by one-way ANOVA gave *p*-values of 0.006, 0.017 and 0.004, respectively. The difference in lanthanum uptake may be due to a number of factors: the thermodynamic stability of the La(XT) complex (log *β*_ML_ = 13.0(3), pM (= –log[La^3+^])§
§pM values were calculated at physiologically relevant conditions of pH 7.4, 100 μM ligand, and 10 μM metal. of 12.0) is greater than that for La(dpp)_3_ (log *β*_ML3_ = 17.44, pM of 5.8) and/or the presence of the phosphinate group in XT may improve the targeting of lanthanum to bone.^31^,^32^

It is also worth noting that consistently higher levels of La^3+^ were detected in the knee section of the femur *versus* the hip and middle sections, suggesting regions of higher bone turnover actively incorporate La^3+^ ions into the bone structure more rapidly. The lanthanum detected in bone was consistently and significantly greater than background levels in untreated SD rats (41.9 ± 12.4 ng g^–1^). The low background levels were less than 5% of detected La^3+^ in treated animals.

Overall, lanthanum is rapidly cleared from the blood with redistribution to bone, as well as certain tissues, predominantly the liver and spleen. This is consistent with previous findings that show the initial uptake of lanthanide elements is in the liver followed by redistribution to the bone.^41^

### Binding thermodynamics of ligands and La^3+^ complexes with hydroxyapatite

Having evidence from previous studies of binding between HAP and La^3+^ in the presence of either Hdpp or H_5_XT,^30^,^31^ we sought to further investigate the nature of this interaction using ITC and standard solution-depletion studies. Previously we used ITC to characterise the thermodynamics of ion-exchange between Ca^2+^ and La^3+^ within synthetic HAP.^37^ Here, we determine the effect of La^3+^ chelation by a ligand on its HAP binding ability and affinity. These titrations were performed first at pH 5 to permit direct comparison with our previous ITC titrations of free metal ion with HAP, and then at pH 7.4.

**Fig. 6:**
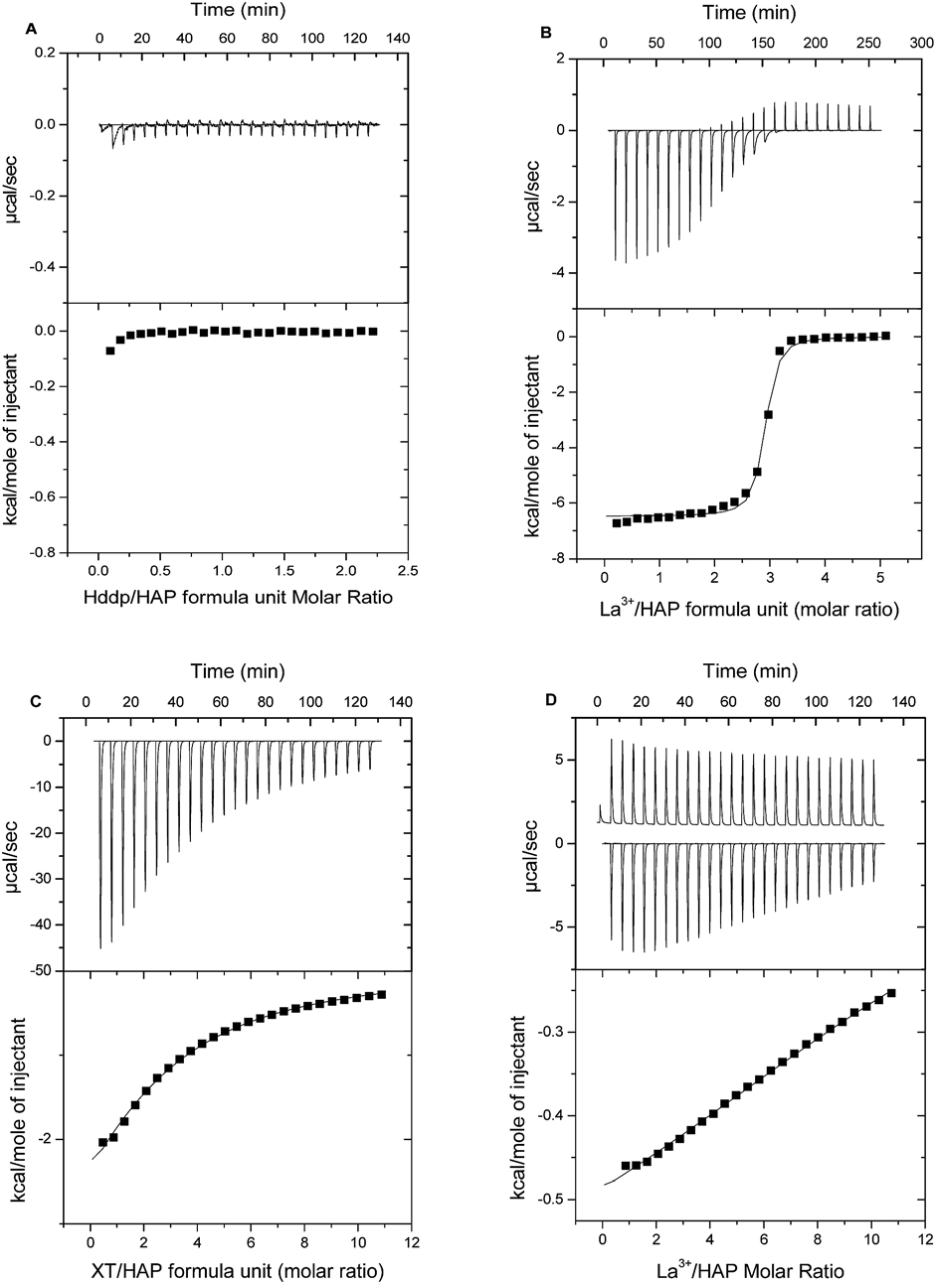
ITC analysis of binding to hydroxyapatite (HAP) at 37 °C, pH 5.0 (100 mM piperazine). (A) (Upper) Raw titration data for 10 μL injections of Hdpp ligand alone (9.8 mM) into the ITC cell containing 0.9 mM formula units of HAP. (Lower) Integrated heat data (points) for the Hdpp into HAP titration. (B) (Upper) Raw titration data for 10 μL injections of La^3+^ (2.5 mM)/Hdpp (10 mM), into the ITC cell containing 0.1 mM formula units of HAP. (Lower) Integrated heat data (points) for titration of La^3+^ + Hdpp into HAP and best fit (line) to a one-site bimolecular–bimolecular binding model. (C) (Upper) Raw titration data for 10 μL injections of 80 mM H_5_XT into the ITC cell containing 1.5 mM formula units of hydroxyapatite. (Lower) Integrated heat data (points) and best fit (line) to a one-site bimolecular–bimolecular binding model. (D) (Upper) Raw titration data for 10 μL injections of La^3+^ (81 mM)/H_5_XT (91 mM), into the ITC cell containing 1.5 mM formula units of HAP. (Lower) Integrated heat data (points) for the La^3+^ + H_5_XT into HAP titration.

Fig. 6A and B show ITC titrations of Hdpp ligand alone and La^3+^ in the presence of Hdpp, respectively, into HAP at pH 5. We do not see any evidence of interaction between the hydroxypyridinone ligand, Hdpp, and HAP in the absence of La^3+^. Titrations of the La^3+^ ion in the presence of Hdpp at pH 5 shows binding of La^3+^ to HAP through displacement of Ca^2+^.

Examination of the speciation of La^3+^ and Hdpp under ITC titration solution conditions facilitates understanding of the observed binding events. The species composition of the titrant solution within the ITC syringe (2.5 mM La^3+^, 10 mM Hdpp) at pH 5 (Fig. S1A†) shows that approximately 35% of lanthanum exists as the free La^3+^ ion, 60% is in the form of [La(dpp)]^2+^ and less than 5% is [La(dpp)_2_]^+^. Following the first 10 μL injection of titrant, most (>95%) of the La^3+^ then exists as the free ion within the ITC cell, with La(dpp)^2+^ comprising the small remaining fraction (Fig. S1B†). After the 25th (final) 10 μL injection, the distribution is 75% La^3+^ and 25% La(dpp)^2+^. Thus, at pH 5, differential heats recorded in each titration essentially represent free La^3+^ ion binding to HAP, and a *K*_a_ value very similar to that determined for La^3+^ alone was therefore recorded by ITC (Table 2).

**Table 2:** Apparent equilibrium constant^*a*^ *K*_a_ for binding of various La^3+^ species^*b*^ to hydroxyapatite at 37 °C

Species	*K*_a_ M^–1^ (pH 5)	*K*_a_ M^–1^ (pH 7.4)
Free La^3+^	2.4 (±0.2) × 10^6^	NA
La^3+^ + Hdpp	1.7 (±0.4) × 10^6^	1.3 (±0.3) × 10^4^
La^3+^ + H_5_XT	<100^*c*^	ND

^*a*^ *K*_a_ values measured by ITC and reported as “apparent” as the differential heat data were fit to a standard one-site bimolecular–bimolecular binding model.

^*b*^ITC results for binding of the free La^3+^ ion to HAP at 37 °C and pH 5 were taken from Cawthray *et al.*^37^ That study did not include studies of the same binding system at pH 7.4 (NA).

^*c*^Following subtraction of controls, binding at pH 5 was too weak to permit precise determination of *K*_a_ at pH 5. No estimate (ND) of *K*_a_ could be obtained for this system at pH 7.4.

At pH 7.4, as at pH 5, no binding is observed for titrations of the Hdpp ligand alone into HAP, allowing direct measurement of binding thermodynamics of La^3+^ and HAP when the Hdpp ligand is present. Though the binding enthalpy is smaller in this case, ITC results for the titration are otherwise similar to those shown in Fig. 6B, and a comparison of the *K*_a_ values recorded is provided in Table 2 along with data for the interaction of the isolated metal with HAP. The *K*_a_ for La^3+^ binding to HAP through Ca^2+^ displacement when the Hdpp ligand is present is approximately two orders of magnitude larger at pH 5 *versus* pH 7.4. Speciation plots (Fig. S1A and B†) again provide an explanation; they show that at the higher pH, very little free La^3+^ ion exists under any condition throughout the titration. As a result, La^3+^ binding to HAP at pH 7.4 must overcome the added energetic barrier of shedding the dpp^–^ ligand.

In contrast to the Hdpp ligand, at pH 5 the phosphinate-EDTA derivative, H_5_XT, was found by ITC to bind HAP in the absence of La^3+^ (Fig. 6C). In the presence of H_5_XT, binding of La^3+^ to HAP is also observed at pH 5 by ITC (Fig. 6D), but is weaker in nature. Regrettably, in this case, the complex contributions from background binding of the ligand to HAP, as well as the high heat of dilution of the ion-loaded ligand, confounded efforts to regress a *K*_d_ value for La^3+^ absorption.

Similar results were obtained at pH 7.4, with the added complication that binding was considerably slower (resulting in significant titration peak broadening) and, for the case of binding of the H_5_XT ligand alone, each titration exhibited an initial endothermic process followed by a slow exothermic event (Fig. S2†). Complex binding of multidentate anionic ligands has been observed before, as evidenced by the 2-site model previously proposed for binding of bisphosphonates to human bone at pH 7.^42^ That system is thought to be comprised of a weak, highly populated site where a phosphonate binds into the bone mineral matrix, and a second higher affinity binding site. Binding at the weaker site results in displacement of one phosphonate group per ligand, with the energy required to release that group contributing to the binding free energy. For the H_5_XT ligand, binding to HAP observed by ITC is complex, but relatively weak overall (apparent *K*_a_ of 1.9 (±0.4) × 10^2^ M^–1^ at pH 5), and therefore more consistent with the characteristics of the weaker site described above. As with weak-binding bisphosphonates, H_5_XT uptake is therefore likely characterized by relatively high rates of desorption.^43^

Whilst the complex energy landscape of the control experiments prevented determination of *K*_a_ values for La^3+^ binding to HAP in the presence of H_5_XT, evidence for weak ion binding to HAP was recorded at pH 5 by ITC. For that case, speciation plots (Fig. S3†) show that no significant fraction of the added lanthanum exists as free La^3+^ either in the ITC syringe or in the ITC cell at any point during the titration. La^3+^ binding to HAP must therefore overcome the energy required both to shed the XT ligand and to displace the bound Ca^2+^. The 1 : 1 La(XT)^2–^ complex is *ca.* 5 to 6 orders of magnitude tighter than the corresponding La(dpp)^2+^ complex.^31^,^32^ As a result, the net La^3+^ binding interaction with HAP is expected to be considerably weaker (∼10^5^ weaker) than recorded for the La^3+^ + Hdpp system, in accordance with the quite weak differential binding data reported in Fig. 6D which indicate that the affinity of the La(XT) complex is comparable to that characterizing exchange of Ca^2+^ with La^3+^ in HAP.

The ITC studies, while further emphasizing the complexity of the systems, do provide an insight into manner in which these two complexes behave *in vivo*. The data clearly shows that Hdpp has no affinity for HAP and – due to the thermodynamic stability of the tris complex – readily releases the metal ion under physiological conditions. This leads to a plasma clearance and tissue biodistribution akin to what one would expect for La^3+^ ions free from a specific chelator.^41^ Conversely H_5_XT incorporates functionality which not only gives a more thermodynamically stable metal complex, but possesses its own binding affinity for bone mineral as evidenced by the ITC data. This explains the subtle differences in the *in vivo* results, in particular the bone biodistribution, and provides a valuable indicator as to the type of design motif that should be targeted in the quest for a new drug.

**Fig. 7:**
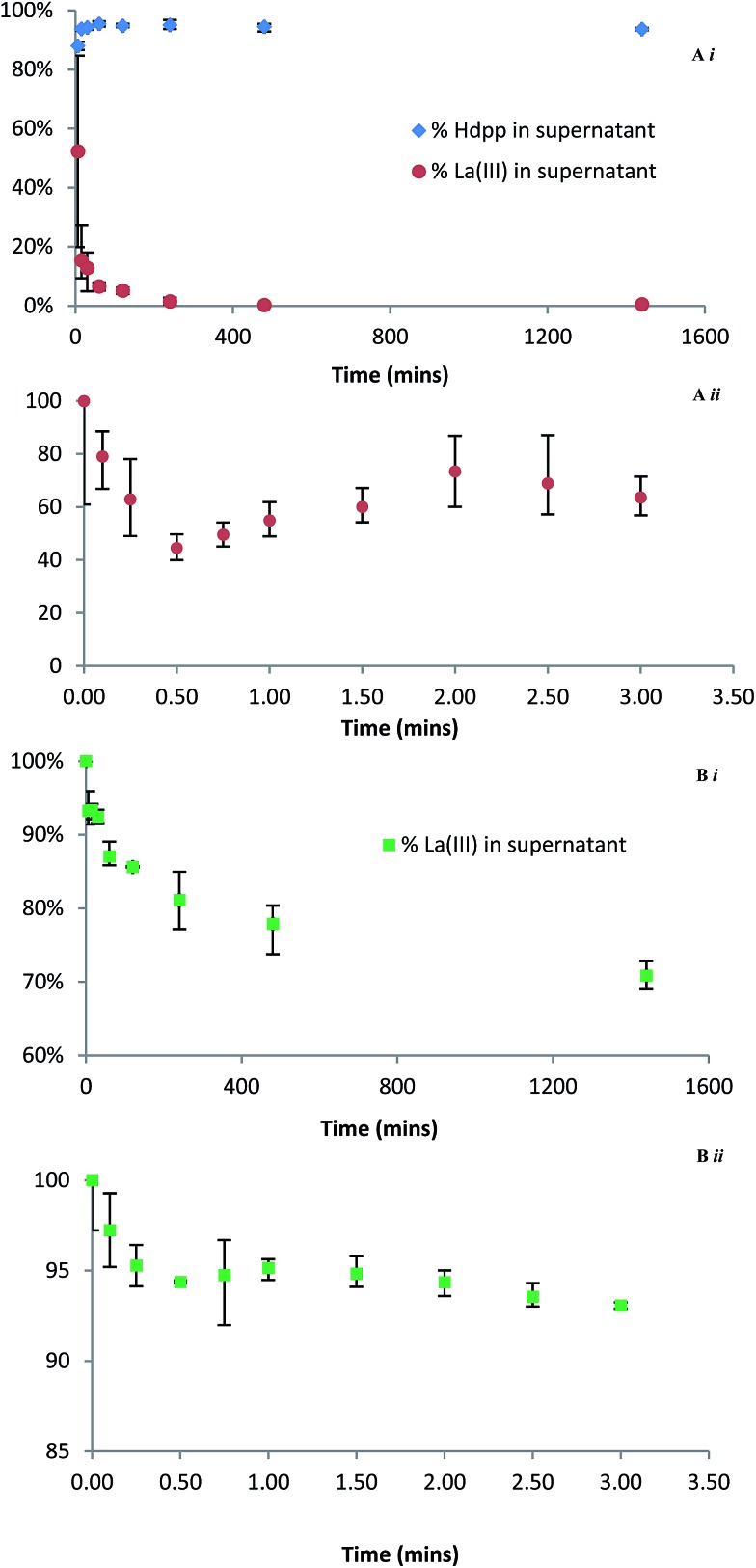
Relative concentration of La^3+^ remaining in the supernatant by ICP-MS after incubation (37 °C, pH 7.4, 220 rpm) with suspended HAP and (A) Hdpp (Ai late time points, Aii early time points) and (B) H_5_XT (Bi late time points, Bii early time points). Ai also shows Hdpp distribution by UV-Vis spectroscopy.

The uptake of La^3+^ by HAP was also investigated in a more traditional batch experiment. The rate of La^3+^ depletion in the supernatant was monitored by incubating solutions containing La^3+^ and either Hdpp or H_5_XT with a suspension of excess HAP at pH 7.4 and 37 °C; the relative distributions of La^3+^ in the supernatant were then followed over regular time intervals by ICP-MS (Fig. 7). In the case of La(dpp)_3_ (Fig. 7Ai), in which distribution of the ligand was also followed by UV-Vis, the data show that, within the error of the experiment, the ligand remains unbound whilst the free metal ion rapidly binds to HAP (greater than 80% in the first 15 minutes). This supports the ITC data that shows that, in the La(dpp)_3_ system, only the metal ion exhibits any affinity for HAP.

In the case of La(XT) (Fig. 7Bi), the ICP-MS data show less than 20% of La^3+^ is bound to HAP after 4 h, and the La^3+^ sorption process is characterized by uptake kinetics that are considerably slower than recorded for the corresponding La(dpp)_3_ system. Unfortunately the distribution of H_5_XT could not be followed by UV-Vis spectroscopy due to the absence of a chromophore within the ligand. Both findings are consistent with the much higher stability La(XT)^2–^ complex (relative to the dominant La(dpp)^2+^ complex in the other system) and the relatively weak net exchange of Ca^2+^ for La^3+^ within HAP when the lanthanum is presented in solution as La(XT)^2–^.

In an effort to elucidate rate constants for the binding of La^3+^ to HAP in the presence of either ligand system, the experiment was repeated with measurements taken at regular early time points. The data show that, for both Hdpp (Fig. 7Aii) and H_5_XT (Fig. 7Bii), there is an initial, rapid association of La^3+^ to HAP in the first 30 seconds, followed by an apparent release of the metal ion, followed by steadier binding consistent with the kinetics of the later time points. This suggests that there are at least two overlapping kinetic processes occurring which are independent of the ligand present. Though this prevents the extraction of meaningful rate constant values, the less pronounced La^3+^ fluctuation in the case of H_5_XT compared to Hdpp is consistent with a ligand system that binds the metal ion more tightly, allowing fewer ions to bind to HAP. A considerably more in depth investigation beyond the scope of this manuscript would be needed in order to determine the cause of this apparent bind-release-bind phenomenon; however, we hypothesize that the presence of two unique Ca^2+^ sites within HAP that possess differing tendencies for exchange with La^3+^ is at the root of the observed occurrence.

Finally, we note that the strength of binding to HAP of the H_5_XT ligand alone is approximately an order of magnitude greater at pH 7.4 than at pH 5. This pH-dependent difference in binding strength is likely due to differences in ligand charge as a result of deprotonation at the higher pH. At pH 5, the ligand exists predominately as H_2_XT^3–^, whereas at pH 7.5 the predominate species is HXT^4–^. This may contribute to the slower La^3+^ uptake kinetics recorded at pH 7.4, less than that recorded for the La(dpp)_3_ system.

There are a number of possible mechanisms that may control the uptake of La^3+^ by HAP: surface adsorption, cation exchange or dissolution and precipitation. We have previously shown that ion exchange reaction between La^3+^ and Ca^2+^ ions of HAP occurs and can be expressed as eqn (1).

1 Ca_10_(PO_4_)_6_(OH)_2_ + *x*La^3+^ → *x*Ca^2+^ + Ca_10–*x*_La_*x*_(PO_4_)_6_(OH)_2_

The use of Hdpp and H_5_XT ligands to mask La^3+^ ion increases the complexity of the system beyond that of simple ion exchange of the metal for Ca^2+^ as seen previously.^37^ This is evident from the *in vitro* ITC analysis and HAP-binding studies where differences for La^3+^-HAP binding were observed depending on the nature of the ligand present. For example, the presence of Hdpp (which does not bind HAP in the absence of metal ion) at pH 7.4 lowers the binding energy of La^3+^ with HAP. In contrast to this, H_5_XT is able to bind HAP in the absence of La^3+^ and also alters the binding energy and kinetics with La^3+^.

The increase in complexity of the system when La^3+^ is introduced in the form of chelate complexes is also evident when the *in vivo* results are compared with *in vitro* studies. The presence of either Hdpp or H_5_XT had a notable influence *in vitro* and *in vivo* on the binding of La^3+^ to HAP as evident in ITC and in the biodistribution of La^3+^ in bone. It is known that, for any La^3+^ complex, the route of administration (oral or intravenous), the quantities used and the chemical speciation of the forms that reach the blood will generates complex differences that complicate any comparisons. This makes comparison of the two reported La^3+^ complexes with the clinically approved lanthanum carbonate. However it is known that La accumulation is time-dependent and longer term studies are currently underway to assess the effects over a longer period of time. Distribution and uptake of lanthanum within bone cannot be accurately determined from the acute study reported here as the time of the bone remodelling cycle falls outside the studies timeline. Therefore, we are currently undertaking a chronic study to assess the bone uptake, distribution within bone and effect on microarchitecture.

## Conclusions

This study examines the lanthanum complexes of two compounds, Hdpp and a phosphinate-EDTA derivative H_5_XT, as potential treatments for bone resorption disorders. We have demonstrated that La^3+^ accumulates in the bone following IV dose of either La(dpp)_3_ or La(XT) with the latter showing slightly higher uptake. These results provide strong evidence that higher denticity chelators, such as H_5_XT, which lead to complexes with greater inherent thermodynamic stability are needed in order to truly influence the *in vivo* behaviour of the lanthanum ions. In addition we have conducted a thorough investigation into the binding kinetics between La^3+^ and hydroxyapatite in the presence of either ligand system, using various techniques and under various conditions, shedding some light onto what is both a fascinating and highly complex binding interaction. This study suggests further *in vivo* experiments are called for to assess their drug candidacies.

## Experimental

### General

High purity water (18.2 MΩ cm, ELGA Purelab Options and ELGA Purelab Ultra) was used in all experiments. All glassware was soaked overnight in HNO_3_ (5%) and thoroughly rinsed with deionized water followed by MQ water to remove any adventitious metal. Lanthanum perchlorate was purchased from Alfa Aesar and used without further purification. Piperazine and hydroxyapatite were purchased from Sigma-Aldrich. The Ca^2+^ and PO_4_^3–^ contents (Ca/P molar ratio of 1.65) for the hydroxyapatite used in this study were determined by ICP-OES and further characterized using TGA, BET analysis and powder XRD as reported previously.^31^,^37^ All metal-ion solutions were prepared the day of use. Lanthanum standard (1000 μg mL^–1^ in 2% HNO_3_) for ICP-MS was purchased from High Purity Standards.

### Synthesis

La(dpp)_3_ and La(XT) were prepared according to previously published methods with slight modifications.^30^–^32^ Briefly, for La(dpp)_3_: commercially available Hdpp (346 mg, 2.49 mmol) and La(NO_3_)_3_·6H_2_O (359 mg, 0.83 mmol) were taken up in 3 mL of deionized water and gently heated and stirred until the ligand had completely dissolved. The pH was very slowly raised with 1 M KOH until a basic pH was obtained. After 3 hours, a white precipitate had formed which was collected by filtration and washed 3 times with cold methanol and dried on a high vacuum overnight. For La(dpp)_3_·3H_2_O: anal. calcd for C_21_H_30_LaN_3_O_9_: C, 41.53; H, 4.98; N, 6.92. Found: C, 41.39; H, 5.00; N, 6.65.

For La(XT): iminodiacetic acid (5.4 g, 40.6 mmol) was suspended in 6 M HCl (10 mL) and stirred and heated to reflux. Hypophosphorous acid (50% w/w, 2.1 mL, 20.3 mmol) was added, followed by the dropwise addition of formaldehyde (37% w/w, 6.4 mL, 80 mmol). After 8 hours, H_5_XT was obtained as the HCl salt as a white precipitate which was collected by filtration and washed with cold methanol followed by cold acetone. H_5_XT·HCl (91 mg, 0.23 mmol) and La(NO_3_)_3_·6H_2_O (100 mg, 0.23 mmol) were waken up in 2 mL of deionized water and stirred. Dropwise addition of 1 M KOH initially caused a white precipitate to form, which gradually dissolved as a basic pH range was obtained. The crude reaction mixture was evaporated, and the residual white solid was taken up in the minimum volume of H_2_O, and precipitated with excess of a 1 : 1 mixture of methanol and acetone. The final product was collected by filtration, washed with acetone, and dried on a high vacuum overnight. For K[La(K·XT)]·3H_2_O: anal. calcd for C_10_H_18_K_2_LaN_2_O_13_P: C, 19.30; H, 2.92; N, 4.50. Found: C, 19.31; H, 2.87; N, 4.45.

### Acid digestion for ICP-MS analysis

Lanthanide concentration for both *in vivo* and *in vitro* studies were determined by ICP-MS analysis by the method reported previously.^30^ In brief, samples were dried (vacuum centrifugation) and dissolved in conc. nitric acid (Optima) and slowly heated to 105 °C over 1 h and maintained at 105 °C for 24 h. Hydrogen peroxide (approximately 2 mL) was added and samples heated at 140 °C for 24 h. Samples were evaporated to dryness at 150 °C before redissolving in 3 mL of 10% nitric acid (with 10 ppm Rh) prior to analysis by ICP-MS.

### General animal studies

All animal study protocols were approved by The University of British Columbia's Animal Care Committee. Female Sprague Dawley (SD) rats (290–320 g body weight) were purchased from Charles River Laboratories (Wilmington, MA, USA).

### Plasma clearance and pharmacokinetics of La(dpp)_3_

Solutions of La(dpp)_3_ were prepared in 10% DMSO and the stability of the complex over time (7 days) monitored by spectrophotometric methods. The solution of La(dpp)_3_ was administered to the animals through cannulae implanted in their jugular veins (*n* = 6). The animals received a dose of 1 mg kg^–1^ at time 0 h and blood samples (0.25 mL were withdrawn at pre-dose, 5, 10, 15, 30 min and 1, 2, 3, 4, 6, 10, 24, 48, 72, 96 and 144 h post-dose). The animals were sacrificed following the last time point and the spleen, liver, kidney, lungs, heart and brain were collected. Lanthanide concentrations were determined by ICP-MS analysis following acid digestion of the samples. The calibration curves were linear in the range of 0.1–100 ng mL^–1^ and 1–50 ng mL^–1^ in the organs and plasma; respectively. The plasma pharmacokinetic parameters were derived by non-compartmental analysis of plasma concentration-time profiles using Phoenix software (Ver. 1.3).

### Tissue distribution of La(dpp)_3_ and La(XT) following multiple doses

Solutions of La(dpp)_3_ and La(XT) (1 mg mL^–1^) were prepared in 10% DMSO and stored at 4 °C. Animals were administered with a dose of 1 mg kg^–1^ once daily for 5 consecutive days by intravenous injection *via* jugular vein port. The animals were sacrificed on the day of the 5th dose and the spleen liver, kidney lungs, heart, brain and bones were collected. Blood samples (0.5 mL) were withdrawn at pre-dose and post-dose. The blood was centrifuged for 10 min at 50 000 rpm at room temperature to obtain the plasma. For organ homogenization, 1 g organ was added to 2 mL normal saline. Bones were cleaned manually of all soft flesh and sectioned into knee, middle, and hip parts using a ceramic blade, and lyophilized prior to normal acid digestion.

### Isothermal titration calorimetry

ITC experiments in the presence of ligand (Hdpp or XT) were carried out in either piperazine (100 mM) pH 5 or HEPES (100 mM) pH 7.4. Titrations were performed by injecting consecutive 10 μL aliquots of ligand (2.5–80 mM) or metal ion/ligand solution (2.5/10 mM for Hdpp and 81/90 mM for XT) into the ITC cell (volume = 1.4 mL) containing hydroxyapatite (0.1–1.5 mM) suspension. Hydroxylapatite suspensions were prepared by adding the required amount of hydroxyapatite to the appropriately buffered solution followed by sonication for 5 minutes. The ITC cell was stirred continuously during the titration. Control experiments (heats of dilution) were performed by titration of ligand or metal ion/ligand solution into buffer containing no hydroxyapatite. Each ITC experiment was performed in triplicate (at least). The error associated with diffusion from the syringe during baseline equilibration, the first injection was only 5 μL, and the associated small heat was not included in the data analysis.

### Hydroxyapatite binding studies

All time points were run in triplicate. Samples containing 10 mg of hydroxyapatite (Sigma-Aldrich) were initially incubated and agitated (37 °C, 220 rpm) with 0.9 mL HEPES buffer (100 mM, pH 7.4) in 1.5 mL Eppendorfs for 24 hours. Stock solutions (1.0 mM) of La(dpp)_3_ and La(XT) were prepared from the premade complexes dissolved in HEPES buffer. At the outset of the experiment, 100 μL of either stock solution was added to a sample and incubated and agitated for a set time. When the time point was reached, the supernatant was carefully removed, taking care not to disturb the residual HAP, and filtered through a 22 micron frit. An accurately weighed aliquot (100 mg) of the supernatant was prepared for ICP-MS analysis using the digestion and evaporation protocol as described for *in vivo* studies. Samples containing La(dpp)_3_ were also analysed by UV-Vis spectroscopy to determine Hdpp concentration. Zero time points were simulated by preparing samples containing no HAP. Background La^3+^ levels were corrected for against samples containing no complex.

## Abbreviations

ANOVA: Analysis of variance
AUC: Area under curve
AUMC: Area under first moment curve
*C*_0_: Initial concentration
Cl: Total body clearance
DMSO: Dimethyl sulfoxide
Dpp: 3-Hydroxy-1,2-dimethylpyridin-4-one
EC_50_: Half maximal effective concentration
EDTA: Ethylenediaminetetraacetic acid
FDA: Food and drug administration
HAP: Hydroxyapatite
HEPES: 4-(2-Hydroxyethyl)-1-piperazineethanesulfonic acid
ICP-MS: Inductively coupled plasma mass spectrometry
ITC: Isothermal titration calorimetry
IV: Intravenous
*K*_el_: Elimination rate constant
Rpm: Revolutions per minute
SD: Sprague Dawley
*T*_1/2_: Half-life
UV-Vis: Ultraviolet-visible
*V*_ss_: Volume of distribution at steady state
XT: Bis[[bis(carboxymethyl)amino]-methyl]phosphinate

## Supplementary Material

Supplementary information
