# Supplementary material for: In vivo study and thermodynamic investigation of two lanthanum complexes, La(dpp)3 and La(XT), for the treatment of bone resorption disorders
Source: Chem Sci. 2015 Aug 3;6(11):6439–47. doi: 10.1039/c5sc01767j (PMC6054118; doi:10.1039/c5sc01767j)
Supplement: Supplementary file 1 [file SC-006-C5SC01767J-s001.pdf]

Electronic Supplementary Material (ESI) for Chemical Science.
This journal is © The Royal Society of Chemistry 2015

Electronic Supplementary Information (ESI) for:

In vivo study and thermodynamic investigation of two lanthanum complexes, $La(dpp)_3$ and La(XT), for the treatment of bone resorption disorders

J. F. Cawthray, †[a] D. M. Weekes, †[b] O. Sivak,[c] A. L. Creagh,[d] F. Ibrahim,[e] M. Iafrate,[b] C. A. Haynes,[d] K. M. Wasan*[a] and C. Orvig*[b]

Fig. S1a: Speciation plot calculated for solutions of La^{3+}-Hdpp under typical conditions employed for ITC. [La^{3+}] = 0.0025M, [Hdpp] = 0.010M

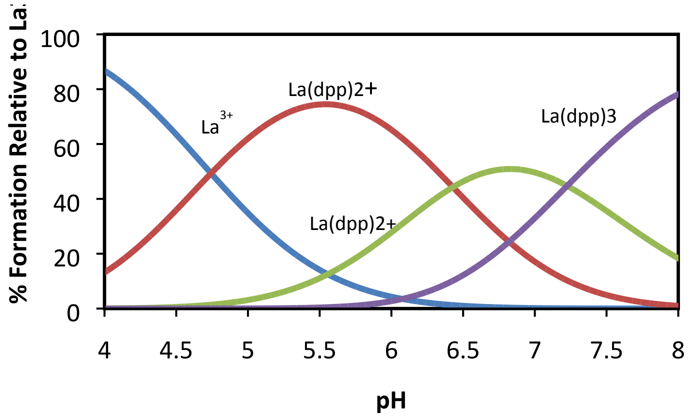

Fig. S1b: Speciation plot calculated for solutions of La^{3+}-Hdpp under typical conditions employed for ITC following first injection of 10 ul. [La^{3+}] = 0.0000177M, [Hdpp] = 0.0000709M

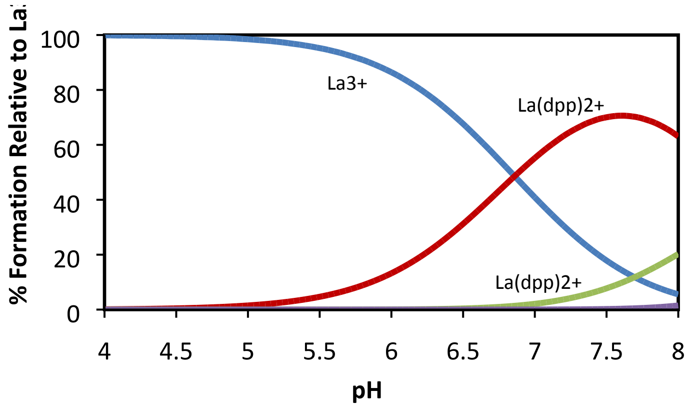

Fig. S2: ITC analysis of H_5XT-hydroxyapatite titration at 37 °C at pH 7.4 (100mM HEPES). (Upper) Raw titration data for 10µL injections of 30.4mM H_5XT^+ into the ITC cell containing 1.1 mM formula units of hydroxyapatite. (Lower) Integrated heat data (points) and best fit (line) to a one-site bimolecular-bimolecular binding model.

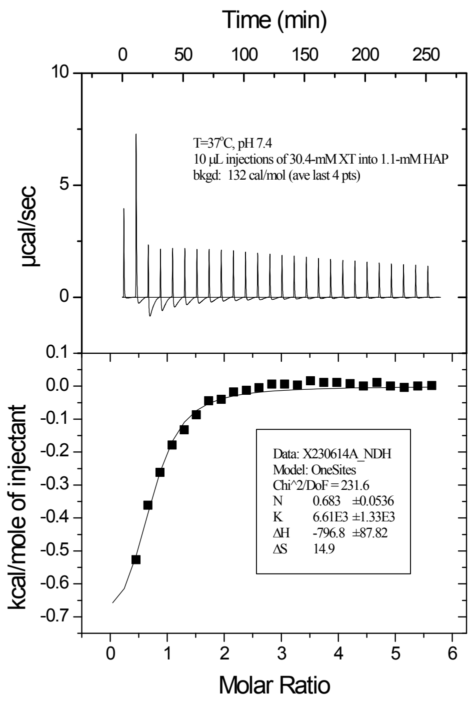

Fig. S3: Speciation plot calculated for solutions of La^{3+}-H_5XT under typical conditions employed for ITC. [La^{3+}] = 0.0025M, [H_5XT] = 0.010M

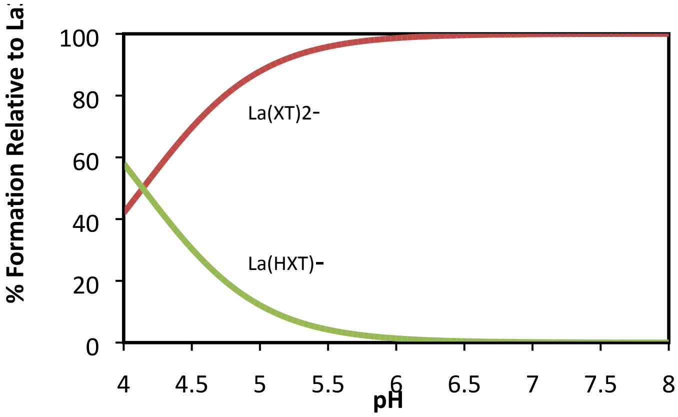

Table S1: Plasma concentrations of La(dpp)3 and LaXT following IV dose of 1mg/kg/day taken at beginning & end of trial (ng/g weight, mean ± SD, n=6).

La(dpp)	t=0	t=end	LaXT	t=0	t=end
	3.8	4971.8		6.9	5843.4
	8.1	4668.4		3.7	6432.4
	6.6	4232.8		9.3	5776.8
	10.0	7201.5		9.5	6337.3
	6.0	6740.2		3.8	5782.3
	6.8	5287.1		7.2	5819.9
**Mean**	6.9	5517.0	**Mean**	6.8	5998.7
SD	2.1	1187.7	SD	2.5	301.6
